# Squamous cell carcinoma of the tonsillar remnant - clinical presentation and oncological outcome

**DOI:** 10.1186/1758-3284-3-4

**Published:** 2011-01-19

**Authors:** Christopher J Skilbeck, Jean-Pierre Jeannon, Mary O'Connell, Peter R Morgan, Ricard Simo

**Affiliations:** 1 Department of Head and Neck Surgery, Royal Marsden Hospital, Fulham Road, London. SW3 6JJ. UK; 2 Department of Otorhinolaryngology, Head and Neck Surgery, Guy's and St Thomas' Hospital NHS Foundation Trust, Great Maze Pond, London. SE1 9RT. UK; 3 Department of Clinical Oncology, Guy's and St Thomas' Hospital NHS Foundation Trust, Great Maze Pond, London. SE1 9RT. UK; 4 Department of Oral Pathology, King's College London & Guy's and St Thomas' Hospital NHS Foundation Trust, Great Maze Pond, London. SE1 9RT. UK

## Abstract

**Background:**

Squamous cell carcinoma (SCC) of the tonsil is the most common malignant tumour of the oropharynx. Paediatric tonsillectomy is one of the most commonly performed procedures in Otorhinolaryngology. SCC of the tonsil remnant (SCCTR) in a previously tonsillectomised patient is rare.

**Methods:**

Retrospective review of patients with SCCTR presenting to the Otorhinolaryngology, Head and Neck Unit January 2000 to December 2007.

**Results:**

Two hundred and fifty patients with tonsil SCC were identified. Ten (4%) of these had previously undergone tonsillectomy in childhood. Nine patients underwent radical treatment including surgery, radiotherapy and in four cases concomitant chemotherapy. Eight patients are alive with no signs of recurrence with follow-up of a minimum of 24 months. One has been lost from follow-up.

**Conclusions:**

Clinicians should be aware that SCC can arise from a tonsillar remnant. SCCTR has similar oncological outcomes as tonsillar tumours.

## Background

Paediatric tonsillectomy is a very common procedure performed for a variety of indications, most commonly to prevent recurrent acute tonsillitis. In England 51,318 tonsillectomies were performed in the year 2005/2006. 58% of these operations were in patients under the age of fifteen [1]. The technique for tonsillectomy has evolved over time. Recent innovations using LASER, powered instruments and diathermy have replaced traditional cold steel methods, although these are still commonly used. It is well known that some techniques will not remove the totality of the tonsil tissue and therefore tonsillar remnant will be left. It is also known that minimal remnants can re-grow due to immunological stimulation [2].

The incidence of pharyngeal cancer in England is 4.0 per 100,000 with 1,339 diagnoses in the year 2000. The most common site of cancer within the pharynx is the palatine tonsil with just over 400 new cases per year in England [3]. However, squamous cell carcinoma of the tonsillar remnant (SCCTR) in a previously tonsillectomised patient is rare and with only one previously documented case[4].

Pharyngeal SCC commonly spreads to the cervical lymph nodes. At the time of presentation, 22% of patients with pharyngeal cancer have cervical metastases [5]. However, 10% of all cervical lymph node metastases present without a known primary site [6]. The majority of unknown primary tumours originate from Waldeyer's ring [7]. Investigation protocols for the patient with cervical lymphadenopathy and an unknown primary may vary but usually comprise, fine needle aspiration cytology with or without ultrasound guidance, cross sectional imaging with Computerised Axial Tomography (CT), Magnetic Resonance Imaging (MRI) or both of head, neck and chest regions and panendoscopy and biopsy of 'at-risk' sites including ipsilateral tonsillectomy, or bilateral when indicated. ^18^Fluorodeoxyglucose Positron Emission Tomography (^18^FDG-PET) may be useful in the localisation of occult primary tumours in patients presenting with metastatic nodal disease in the head and neck, although it is not yet fully utilised [8].

The incidence and biological behaviour of tonsillar SCC has been well established and their patterns of spread and response to conventional treatments has have also been extensively reported in the literature [9]. However, the incidence, biological behaviour, patterns of spread and outcomes to treatment of SCCTR have not been previously reported in the oncological literature.

The aim of this paper is to analyse the incidence, clinical presentation, management strategies and clinical outcomes of previously tonsillectomised patients diagnosed with SCCTR.

## Subjects and Methods

A retrospective review of 10 patients with SCC of the tonsillar remnant presenting to the Otorhinolaryngology, Head and Neck Unit at Guy's and St Thomas' NHS Foundation Trust from January 2000 to December 2007 was performed. All patients were seen in the Head and Neck Oncology Unit by the senior authors and underwent a standard protocol of investigation following The British Association of Otorhinolaryngology Head and Neck Surgery Guidelines for the management of head and neck cancer [10]. This includes clinical examination with fibreoptic nasendoscopy, CT of head, neck and chest, MRI scanning where appropriate and rigid endoscopy with biopsies of nasopharynx, ipsilateral tonsil, tongue base and any mucosal abnormality of larynx and pharynx [10].

All patients were discussed at the multidisciplinary head and neck oncology tumour board meeting before their treatment was advised. All patients with Stage I and Stage II disease had single modality treatment and all patients with Stage III and Stage IV disease were treated by combined modality treatment protocols according to our standard operational policy. The oncological stage of their disease was classified according to the International Union Against Cancer (UICC) TNM classification of Malignant Tumours (6^th ^edition) [11].

## Results

From January 2000 to December 2007, 251 patients with squamous cell carcinoma of the tonsil were diagnosed at the Head & Neck Unit at Guy's and St Thomas' Hospital. Ten of these patients (4%) had tonsillectomy performed in childhood. There were seven males and three females with a median age of 56.5 years (range 43-74 years) and a mean of 58.1 years. Two patients were Stage II and 8 Stage IV. In five (50%) patients the index tumour was identified on clinical examination. In the remaining 5 there was no obvious index tumour on clinical examination. They were investigated following an occult primary protocol.

In one patient the histology of the tonsil demonstrated only carcinoma in-situ in the presence of metastatic disease in the ipsilateral lymph nodes. No invasive carcinoma was found in a subsequent wide local excision.

One patient with T_3 _N_3 _M_1 _was treated with palliative intent and the remainder underwent radical planned combined modality treatment. Surgery to the primary site included excision biopsy of the tonsillar remnant in six patients, and trans-oral Potassium Titanyl Phosphate (KTP) LASER excision in three cases. One patient had only an incisional biopsy of the remnant due to the large size of the tumour.

Five patients underwent modified radical neck dissection, one underwent bilateral modified radical neck dissection and one underwent a radical neck dissection. One underwent an excision biopsy of the metastatic cervical lymph node prior to referral. Nine patients received radical radiotherapy to the primary site and neck (65 Gray in 30 fractions over 6 weeks) and in addition four patients received concomitant Cisplatin chemotherapy (75 mg/m^2 ^for 3 cycles). Eight patients are alive with no evidence of disease at a minimum follow-up of 24 months and a maximum of 10 years. One patient died within 2 months of diagnosis as she had untreatable disease and one patient failed to attend follow-up after 12 months.

The patient's demographic data, presenting symptoms, TNM staging, treatment modality and Outcome are summarized in Table 1.

**Table 1:** showing results with presenting symptoms, stage of disease, management and outcome.

Age at diagnosis (years)	Gender	Presenting symptoms	TNM stage	Management	Follow-up (months)	Outcome
43	M	Neck Mass Unknown 1°	T_1_N_3_M_0_	Excision Biopsy RND Radiotherapy	120	Alive

67	F	Neck Mass	T_3_N_3_M_1_	Palliative care	2	Deceased

57	M	Sore throat Neck Mass	T_4_N_2c_M_0_	KTP Bilateral MRND (type I+II) Radiotherapy Cisplatin Chemotherapy	Lost after 12	Alive

56	F	Neck Mass Unknown 1°	T_is_N_2a_M_0_	KTP MRND (type I) Radiotherapy	72	Alive

74	M	Neck Mass Unknown 1°	T_1_N_1_M_0_	Radiotherapy	84	Alive

47	M	Neck Mass Tonsil Mass	T_3_N_3_M_0_	KTP MRND (type I) Radiotherapy	60	Alive

67	F	Sore throat	T_2_N_0_M_0_	Excision Biopsy Radiotherapy	60	Alive

56	M	Neck Mass Unknown 1°	T_1_N_2b_M_0_	MRND Radiotherapy Cisplatin Chemotherapy	68	Alive

50	M	Neck Mass Unknown 1°	T_2_N_2a_M_0_	MRND Radiotherapy Cisplatin Chemotherapy	48	Alive

64	M	Sore throat Neck Mass	T_2_N_3_M_0_	Radiotherapy Cisplatin Chemotherapy Staged MRND	24	Alive

MRND - modified radical neck dissection

RND - radical neck dissection

## Discussion

Tonsillectomy is one of the most commonly performed operations by Otorhinolaryngologists. Techniques for tonsillectomy have evolved and there is a possibility of residual tonsil tissue being left *in situ *as a tonsillar remnant. In this series all patients had their tonsils removed in the 1950's and 1960's when guillotine tonsillectomy was particularly popular. Tonsillectomy is often performed by trainee surgeons. It is possible that the less experienced surgeon might inadvertently leave tonsil tissue in the oropharynx which might account for the presence of remnant. It is also possible for the lymphoid tissue in Waldeyer's ring to re-proliferate due to immunological stimulation either in the palatine tonsil or as an expansion of the lingual tonsil[2].

**Figure 1:**
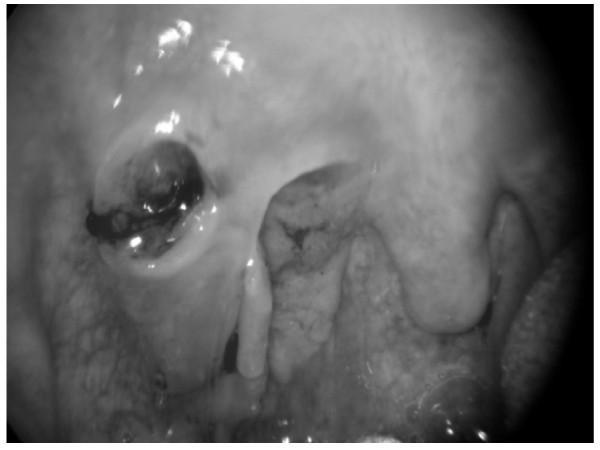
**showing T_2 _primary squamous cell carcinoma of the right tonsillar fossa**. The epicentre of the tumour was found to be in the tonsillar remnant and it ulcerated through the tonsillar pillar.

SCC of the tonsil is the most common malignant tumour of the pharynx accounting for 50% of tumours in this site [10]. The incidence in our unit which covers a population of 1.5 million, is comparable to previously published data [3]. SCCTR has only been previously described as a single case report and as such there is no quoted incidence. Patients with SCCTR may present with symptoms and signs of local, regional or distant metastatic disease. In this series, three patients presented with a sore throat, one with a tonsillar mass (see figure 1) and nine of the patients complained of a lump in the neck. Five (50%) patients had no obvious site of primary tumour when initially seen in clinic.

All patients with suspected head and neck malignancy should have a full otorhinolaryngological assessment and examination. It is important to establish if any previous surgery has been performed, including the age of tonsillectomy. Clinicians should be aware that previous tonsillectomy does not preclude the possibility of a tonsillar remnant and this harbouring SCC. Therefore, a high index of clinical suspicion should be exercised in patients who had previous tonsillectomies and present with potentially occult primary SCC of the head and neck region.

**Figure 2:**
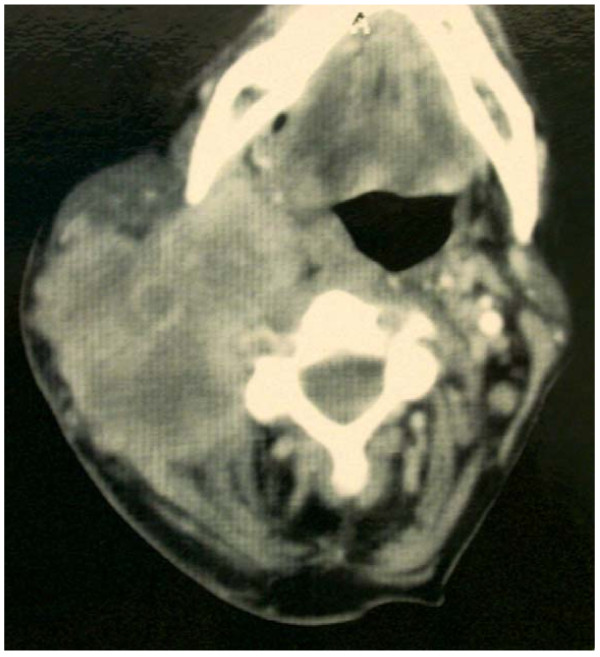
**showing axial CT scan of necrotic right cervical lymphadenopathy**.

Patients who present with a clinically obvious tonsillar mass should undergo examination and biopsy under general anaesthetic. The presence of malignant lateral cervical lymphadenopathy in the absence of an obvious index tumour represents a challenging diagnostic and management dilemma [12,13]. In patients who have no identifiable primary site a systematic approach with an investigation protocol is mandatory. In our unit we follow a validated protocol; namely the BAO-HNS guidelines for management of head and neck cancer. This includes a full otorhinolaryngological examination with fibreoptic nasendoscopy, fine needle aspiration cytology of the cervical lymphadenopathy and cross sectional imaging with CT scanning (see figure 2) and MRI scanning where appropriate. This is followed by upper aero-digestive tract rigid endoscopy with biopsies of the post-nasal space, tongue base, pyriform sinus, ipsilateral tonsillectomy and biopsies of any suspicious focal lesions [10]. Following this diagnostic algorithm up to 95% of primary tumours will be found, and in 33% of cases the index tumour will be located in the tonsil or tongue base [14]. In patients who have undergone previous tonsillectomy the index tumour may be found in the tonsillar remnant and this is the case in 50% of our cohort.

In our unit, radiological investigation for staging head and neck cancer includes ultrasound (US), CT and MRI when assessing soft tissue involvement. Ideally, diagnostic imaging such as CT or MRI, should be performed before biopsy in an attempt to identify the index tumour and allow adequate staging of the disease [13]. Further radiological imaging including ^18^FDG-PET scans may be useful in a certain subset of patients but its usefulness, when available, is yet to be proven especially if the index tumour is smaller than 1 cm in diameter [15]. In our series, all patients with an occult primary were assessed using our standard diagnostic protocol and ^18^FDG-PET was not utilised.

**Figure 3:**
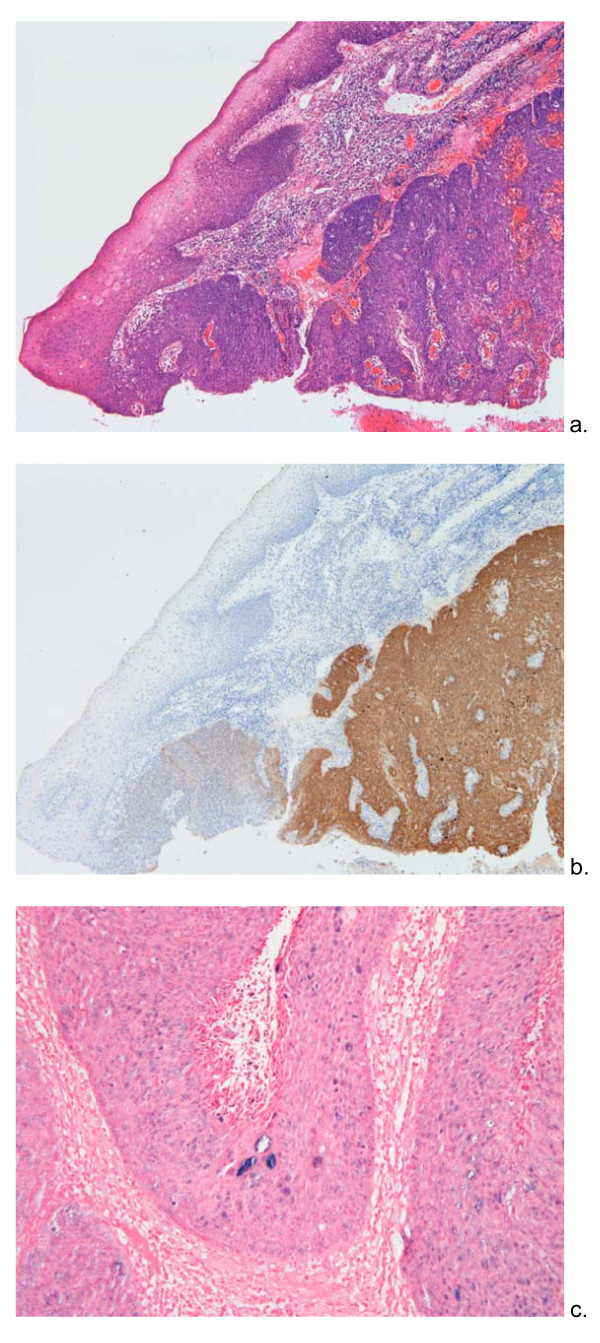
**the histological appearance of one of the cases, a poorly differentiated squamous cell carcinoma stained with Haematoxylin & Eosin**. a: The field shows the border between normal epithelium and poorly differentiated squamous cell carcinoma. b: the equivalent field in an adjacent section showing immunohistochemical staining for p16, a surrogate marker for HPV. c: a higher power view of an adjacent section demonstrating the presence of high-risk HPV by in-situ hybridisation. The viral DNA is stained blue.

Although the histological characteristics and aetiological factors are beyond the scope of the present study, all the tumours were typical of tonsillar carcinomas: poorly differentiated squamous cell carcinomas, including some basaloid squamous types. Figure 3a shows an example, depicting the border between normal pharyngeal epithelium and carcinoma. The period of review predated the routine staining for HPV in tonsillar tumours but there is no reason to suppose that SCCTR differ from conventional tonsillar carcinomas. Immunohistochemical staining for p16, whose up-regulation is a marker for transcriptionally active HPV, was selective for the carcinoma in the same case, normal epithelium being negative (Figure 3b). In-situ hybridisation confirmed the presence of high risk HPV in an adjacent section (Figure 3c).

Oropharyngeal carcinoma presents with clinically or radiologically positive metastatic neck disease in approximately 50% of cases [16]. Therefore, combined modality therapy is considered to treat the primary site and nodal disease. There is controversy on how this tumour site should be managed; primary surgical resection and neck dissection followed by post operative radiotherapy or to offer organ preservation. Our current management strategy has evolved over the past few years in line with current evidence based protocols. We have therefore adopted an organ preservation protocol involving chemo-radiation followed by staged neck dissection in stage III and IV disease and radiotherapy for stage I and II disease with re-evaluation and salvage surgery as appropriate. This protocol was introduced due to previously published work which showed adverse results due to radiotherapy delays following surgery [17]. The therapeutic strategy for patients with SCCTR will depend on the stage of the disease at diagnosis. Patients with proven metastatic neck disease should undergo a combined treatment modality [18]. In our series, combined treatment with surgery and radiotherapy was employed in all patients deemed curable. Surgery to the index tumour consisted of wide excision using a cold steel technique or KTP LASER. The neck disease was treated with either neck dissection combined with radical radiotherapy or radical radiotherapy alone. Four patients received chemotherapy in addition. Using this treatment protocol should provide cure rates of up to 70% at 5 years [19,20]. The prognosis of patients with SCCTR may exceed this. In our series, the 2 year disease free survival is 89% and for five years is 83%. Our series, although limited, represents the first report of the incidence, clinical presentation, management and clinical outcomes of patients with SCCTR. It highlights the importance of this diagnosis, which could be considered as a clinical sub-group within SCC of the tonsil. It also advises clinicians dealing with head and neck cancer of the need for thorough clinical evaluation and the possibility of tonsil biopsy when investigating patients presenting with an unknown primary tumour despite childhood tonsillectomy. The management strategy for patients with SCCTR should be the same as those as those patients with primary SCC of the tonsil and the outcomes for these patients appear to be similar to the published data for SCC of the tonsil.

In conclusion, SCC of the tonsillar remnant could be considered as a clinical sub-group within SCC of the palatine tonsil and clinicians should be aware that carcinomas could arise from a tonsillar remnant. A tonsil biopsy should be performed when investigating a patient with an unknown primary tumour, despite childhood tonsillectomy and a normal appearance of the tonsillar remnant. Tonsillar remnant carcinomas appear to have similar oncological outcomes as tonsillar tumours.

## Competing interests

The authors declare that they have no competing interests.

## Authors' contributions

RS conceived the study. RS, J-PJ, MO'C and CS participated in the design. PM provided advice and guidance on the pathological aspects of the paper. CS and RS prepared the manuscript. All authors read and approved the final manuscript.
